# Alteration of Androgen Receptor Protein Stability by Triptolide in LNCaP Cells

**DOI:** 10.3390/medicina54030039

**Published:** 2018-05-30

**Authors:** Wei Li, Bi-De Liu, Kai Liao, Yong Liu, Zi-Jin Wan, Yu-Fen Dong, Qian-Qian Cao, Qian Zhu, Xiao Gu

**Affiliations:** 1 Translational Medicine Research Institute, College of Medicine, Yangzhou University, Yangzhou 225001, China; liaokai@yzu.edu.cn (K.L.); wanzijin@yzu.edu.cn (Z.-J.W.); dongyoufen@yzu.edu.cn (Y.-F.D.); caoqianqian@yzu.edu.cn (Q.-Q.C.); zhuqian@yzu.edu.cn (Q.Z.); 2 Jiangsu Key Laboratory of Integrated Traditional Chinese and Western Medicine for Prevention and Treatment of Senile Diseases, Yangzhou 225001, China; 3 People’s Hospital of Xinjiang Uygur Autonomous Region, Urumqi 830001, China; gp_lbd@xjrmyy.com; 4 School of Life Science and Medicine, Dalian University of Technology, Panjin 124221, China; yliu@dlut.edu.cn; 5 College of Clinical Medicine, Yangzhou University, Yangzhou 225001, China

**Keywords:** triptolide, androgen receptor, calpain, protein stability

## Abstract

*Background and Objective:* Although triptolide was effective for prostate cancer (PCa), the mechanism is still unclear. Androgen receptor (AR) plays a large role in the development and progression of PCa, even after castration. The present study aimed at investigating the effects of triptolide on AR protein stability and the possible mechanism. *Materials and Methods:* By blocking protein synthesis with cycloheximide (CHX), the effect of triptolide on AR protein stability was investigated with western blot assay. The potential role of calpains in triptolide reduced AR protein stability was investigated with calpain inhibitor and Ca^2+^ chelator. *Results:* Triptolide down-regulated AR protein level when protein synthesis was blocked by CHX, demonstrating the decrease of AR protein stability. The AR protein level was restored when the cells were co-treated with triptolide and calpain inhibitor or Ca^2+^ chelator, indicating the important role of calpains. *Conclusions:* The results indicate that triptolide can activate calpain via promoting intracellular Ca^2+^ accumulation, and thus decrease the stability of AR protein, subsequently resulting in the breakdown of the AR protein in LNCaP cells. This work provides an experimental basis and evidence to elucidate the anti-PCa mechanisms of triptolide.

## 1. Introduction

Prostate cancer (PCa) is one of the most common malignancies in the male urinary system [1,2]. Both androgen and androgen receptor (AR) play a crucial role in the development and progression of PCa, and androgen deprivation therapy is typically employed as a first-line treatment for PCa patients to impede the AR signaling in metastasis PCa [3,4]. However, AR can be activated despite androgen deprivation therapy in AR-positive PCa via an increased sensitivity of AR to low concentrations of androgen, and overexpression of AR has been suggested to be associated with the development of androgen resistance [5,6]. The importance of AR and its up-regulation in progressively worse stages of PCa has called for the development of novel agents involving AR-targeted drugs that will disrupt the AR signaling pathway [4].

Triptolide is the primary active component of a traditional Chinese medicine, *Tripterygium wilfordii* Hook F (TWHF). Its prodrug, PG490-88, is experimentally effective for non-small cell lung carcinoma, fibrosarcoma, and colon carcinoma cells, and has been evaluated in phase I clinical trial for its anticancer activity [7]. We and others have reported the anti-PCa activity of triptolide [8,9], while the mechanism is still under investigation. Some current studies have shown that the anti-PCa effects of triptolide may be associated with its modulating effects on AR. Huang et al. showed both AR mRNA and protein levels were down-regulated by triptolide treatment [10]. Our previous investigation showed that the decreased mRNA expression by triptolide could be attributed to −965/+265 bp of the AR promoter region, and NFκB might contribute to this transcriptional regulation [11].

Post-transcriptional regulation also plays an important role in the regulation of AR activity [12]. AR has a relatively short half-life, indicating that the effects are regulated by systematic protein activation and degradation. Thus, AR stabilization may be related to activation of the AR signaling pathway [13]. Calpains have been suggested to be responsible for AR protein degradation [14,15,16]. Calpains are a family of calcium-activated, cysteine protease. Among the family, calpain-1 and calpain-2 are ubiquitously distributed. There is also an endogenous inhibitor, calpastatin, that blocks the calpain activity [17]. The fact that triptolide induced accumulation of Ca^2+^ in some cells [18,19] prompts us to consider whether the effect of triptolide on AR is related to the regulation of AR protein stability via calpains and is responsible for its anti-PCa activity.

In the present study, the effects of triptolide on AR protein stability and the possible mechanism were investigated. The results showed that triptolide decreased AR protein stability in LNCaP cells via calpain activation. Interestingly, the regulation of AR degradation by triptolide cannot be via altering the calpain protein levels, but via the promotion of Ca^2+^ accumulation in the cells.

## 2. Materials and Methods

### 2.1. Chemicals

Triptolide was purchased from Sichuan Weikeqi-Biotech Company (Chengdu, China); Cycloheximide (CHX) was purchased from Dalian Meilun Biotechnology (Dalian, China); Probenecid was purchased from Shanghai Aladdin Bio-Chem Technology Co., Ltd. (Shanghai, China); A23187 was purchased from BioVision, Inc. (Milpitas, CA, USA), BAPTA-AM was purchased from Selleck Chemicals (Houston, TX, USA); ALLM was purchased from ApexBio Technology (Houston, TX, USA); FLUO-3 AM was purchased from KeyGEN Biotech (Nangjing, China); Phenylmethylsulfonyl fluoride (PMSF) was purchased Sangon Biotech Co., Ltd. (Shanghai, China).

### 2.2. Cell Culture

LNCaP cells were purchased from the Cell Bank of Type Culture Collection of Chinese Academy of Sciences and cultured in RPMI-1640 media (GE Healthcare Life Sciences HyClone laboratories, Logan, UT, USA) with 10% fetal bovine serum (FBS, Clark Bioscience, Richmond, VA, USA).

To detect the effect of triptolide on cell growth, LNCaP were plated on 96-well plate (1.5 × 10^5^/well) and treated with triptolide (100 nM). The viable cells were detected with SRB assay [8].

To detect the AR protein level, after being plated on 6-well plates, LNCaP cells were pre-treated with CHX (20 ng/mL) for 2 h and then triptolide (100 nM) was added to the media. After co-treatment with CHX and triptolide for 24 h, the cells were lysed with RIPA lysis buffer (Beyotime Biotechnology, Haimen, Jiangsu, China) and the protein concentration was detected with BCA kit (Beyotime Biotechnology, Haimen, Jiangsu, China).

To detect the role of calpain in triptolide mediated AR degradation, calpain inhibitor ALLM and calcium chelator BAPTA-AM were used. After co-treatment with triptolide and ALLM (50 μM and 100 μM), PMSF (100 μM) or BAPTA-AM (10 μM) for 24 h, the cells were lysed with RIPA lysis buffer (Beyotime Biotechnology, Haimen, Jiangsu, China) and the protein concentration was detected with BCA kit (Beyotime Biotechnology, Haimen, Jiangsu, China).

### 2.3. Detection of Calcium Concentrations

LNCaP cells were plated on 6-well plates and treated with triptolide (100 nM) for 24 h. Cells were harvested after trypsin digestion and then loaded with Fluo-3 AM (4 μM) for 30 min. Fluo-3 AM was solved in HBSS buffer containing pluronic (2.5 mM), probenecid (0.05%) and FBS (1%). The cells were washed with HBSS buffer (containing probenecid (0.05%) and FBS (1%)) twice and then incubated at 37 °C for 30 min. The intensity of fluorescence (excitation, 485 nm; emission, 525 nm) was read with a microplate reader (Biotech, Winooski, VT, USA). The fluorescence intensity was normalized by cell protein concentration.

### 2.4. Western Blot

Thirty μg proteins were subjected to SDS-PAGE, and the proteins on gels were transferred to PVDF membranes. The membranes were blocked with 5% skim milk, and incubated with anti-AR (1:1000, Cell Signaling Technologies, Inc., Danvers, MA, USA), anti-calpain-1 (1:1000, Thermo Fisher Scientific, Waltham, MA, USA), anti-calpain-2 (1:1000, Thermo Fisher Scientific, Waltham, MA, USA), anti-calpastatin (1:1000, Santa Cruz Biotechnology, Dallas, TX, USA) or GAPDH (1:1000, KeyGEN Biotech, Nangjing, China), then with a horseradish peroxidase-conjugated secondary antibody (1:5000, ZSGB-BIO, Beijing, China). The proteins were visualized using an ECL Western blot kit (Beyotime Biotechnology, Haimen, Jiangsu, China) and document with FluoChem E imaging systems (ProteinSmiple, San Jose, CA, USA).

### 2.5. Statistical Analysis

All experiments were conducted in triplicate. Results were expressed as means ± SD. Statistical analyses were performed with Student’s t-test for comparison of differences between two groups, or ANOVA analysis for comparison of differences of three or more groups. Differences were considered statistically significant when *p* < 0.05.

## 3. Results

### 3.1. Triptolide Decreased the Stability of AR Protein

**Figure 1:**
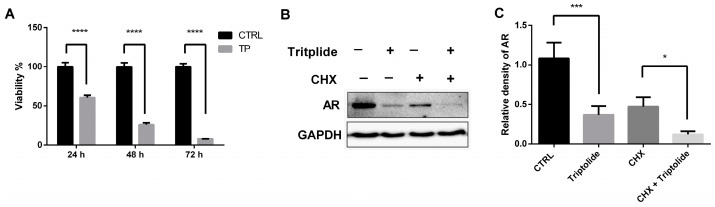
Triptolide down-regulated AR protein level in the presence of CHX. (**A**) The viability of LNCaP Cells were detected with SRB assay after the cells were treated with triptolide (TP, 100 nM); (**B**) LNCaP cells were co-treated with CHX (20 ng/mL) and triptolide (100 nM) for 24 h, and the AR protein level was analyzed by western blot assay; (**C**) The relative density of AR (normalized with GAPDH in western blot assay). (**** *p* < 0.0001, *** *p* < 0.001, * *p* < 0.05).

To investigate whether triptolide can regulate the protein stability of AR, the protein synthesis inhibitor CHX was employed. The results showed that triptolide down-regulated AR protein level in parallel with the decrease of the cell viability after 24-h treatment (Figure 1). In addition, when the protein synthesis was blocked by CHX, the AR protein level was also down-regulated (Figure 1B). These results indicated that triptolide regulated AR protein stability in LNCaP cells.

### 3.2. The Blocking of AR Protein Down-Regulation by Calpain Inhibitor, ALLM

**Figure 2:**
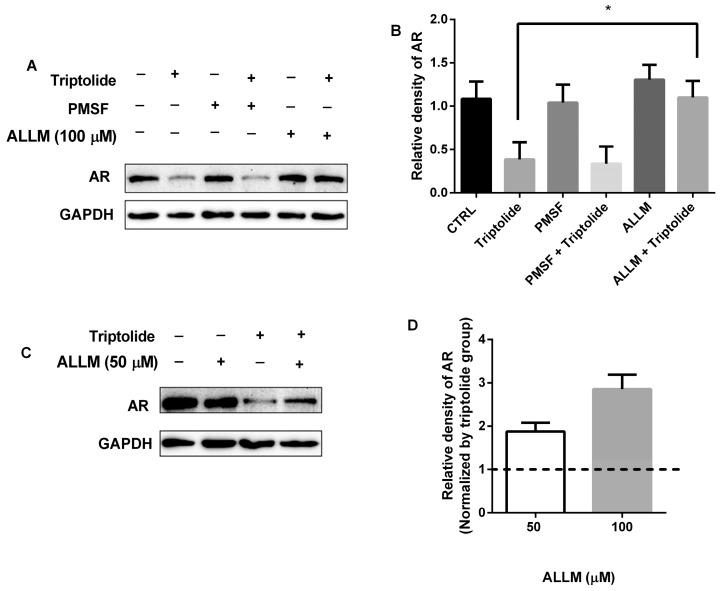
ALLM blocked the AR down-regulation in the presence of triptolide. (**A**) LNCaP cells were co-treated with triptolide (100 nM) and ALLM (100 μM) or PMSF (100 μM) for 24 h, the AR protein level was analyzed by western blot assay; (**B**) The relative density of AR (normalized with GAPDH in western blot assay) after LNCaP cells were co-treated with triptolide (100 nM) and ALLM (100 μM) or PMSF (100 μM) for 24 h; (**C**) The western bolt assay of AR protein levels in LNCaP cells after the cells were co-treated with triptolide (100 nM) and ALLM (50 μM); (**D**) The relative density of AR (normalized with triptolide) after the cells were co-treated with triptolide (100 nM) and ALLM (50 and 100 μM). (* *p* < 0.05).

Proteases play important physiological role in regulatory mechanisms of other proteins by determining their lifetime. To determine whether the proteases involved in triptolide regulated AR down-regulation, protease inhibitor PMSF was first used. PMSF is typically an irreversible inhibitor of serine and cysteine proteases and reacts with common proteases. Although PMSF reacts with some cysteine proteases such as papain, it could not inhibit the activity of calpain effectively [20]. We found that PMSF failed to inhibit AR down-regulation caused by triptolide (Figure 2A). However, in the presence of ALLM, a calpain inhibitor, the down-regulated AR protein caused by triptolide was restored (Figure 2A,C). In addition, with the increase of ALLM concentration (50 and 100 μM), the level of AR protein significantly recovered (Figure 2A,C). Though the recovered AR levels were higher in 100 μM ALLM group compared with those of 50 μM group (2.9 fold vs. 1.9 fold), no significant differences were found between these two concentration groups (Figure 2D). These results indicate that calpain activation plays an essential role in the down-regulation of AR protein by triptolide.

### 3.3. No Effects of Triptolide on the Expression Levels of Calpains or Calpastatin Protein

**Figure 3:**
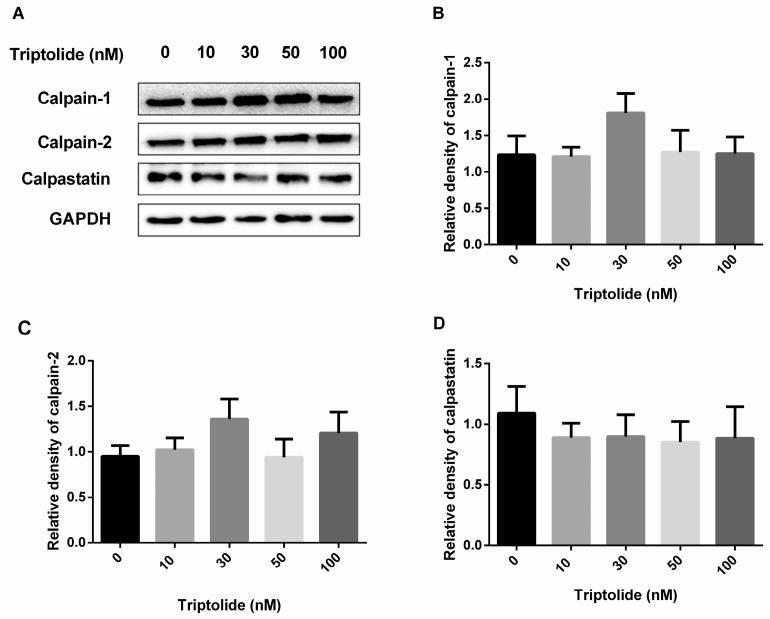
Protein level of calpain-1, calpain-2 and calpastatin. (**A**) After LNCaP cells were treated with triptolide (0, 10, 30, 50 and 100 nM), the calpain-1, calpain-2 and calpastatin were detected with western blot assay; (**B**) The relative density of calpain-1 (normalized with GAPDH in western blot assay) in LNCaP cells after triptolide treatment; (**C**) The relative density of calpain-2 (normalized with GAPDH in western blot assay) in LNCaP cells after triptolide treatment; (**D**) The relative density of calpastatin (normalized with GAPDH in western blot assay) in LNCaP cells after triptolide treatment.

The influence on protein functions may be via the alteration of protein amounts or activity. In view of the essential role of calpain in the regulation of AR protein stability mediated by triptolide, the effects of triptolide on the expression of calpains and their endogenous inhibitory protein were determined first. After triptolide treatment, the protein levels of calpain-1, calpain-2 and their endogenous inhibitor, calpastatin, were determined by western blot assay. None of the three protein levels was significantly altered by triptolide (Figure 3). The result suggests that the calpain-dependent AR down-regulation caused by triptolide could not result from the alteration of calpains or calpastatin protein levels.

### 3.4. Increased Ca^2+^ Level in Triptolide Treated LNCaP Cells

**Figure 4:**
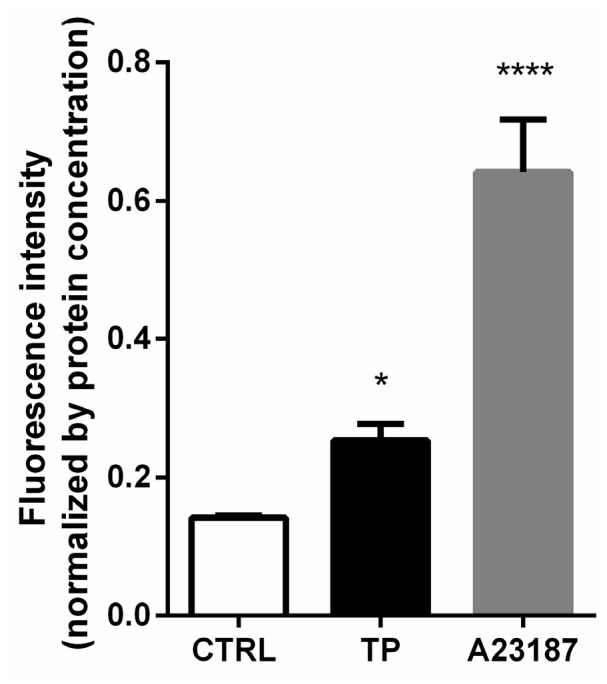
Ca^2+^ level after triptolide or A23187 treatment in LNCaP cells. LNCaP cells were treated with triptolide (100 nM) or A23187 (20 μΜ) for 24 h and loaded with Fluo-3 AM. The fluorescence intense was detected and normalized by cell protein concentration. (**** *p* < 0.0001, * *p* < 0.05).

The analysis of negative data of calpains expression directed our focus toward calcium-triggered events. Calcium influx is known to be central in calpain activation. Calpain has been found to be regulated by calcium influx [21]. Since the activities of calpains depend on the Ca^2+^ levels within the cells, we further investigated the effects of triptolide on cytosolic Ca^2+^ levels. The Ca^2+^ level was determined using FLUO-3 AM. The data showed that, after triptolide treatment, the level of Ca^2+^ in the cells treated by triptolide was significantly higher than the control group, indicating that triptolide could promote Ca^2+^ accumulation in cells and elevate intracellular Ca^2+^ (Figure 4). The result implies that the elevation of Ca^2+^ level after triptolide treatment may be responsible for calpain activation.

### 3.5. The Effects of Calcium Regulator on AR Protein Down-Regulation by Triptolide

**Figure 5:**
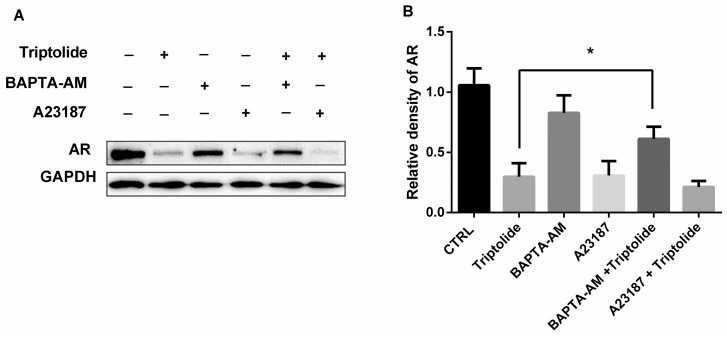
BAPTA-AM blocked the AR down-regulation in the presence of triptolide. LNCaP cells were treated with triptolide (100 nM) in combination with BAPTA-AM (10 μM) or A23187 (20 μM). (**A**) The AR protein level was analyzed with western blot assay; (**B**) The relative density of AR (normalized with GAPDH in western blot assay). (* *p* < 0.05).

To identify whether Ca^2+^ accumulation was responsible for AR protein down-regulation induced by triptolide, both calcium chelator BAPTA-AM (10 μM) and calcium ionophore A23187 were used to explore the role of intracellular Ca^2+^ in AR protein down-regulation induced by triptolide. The data showed that calcium chelator BAPTA-AM recovered the protein level of AR that was decreased by triptolide (Figure 5). Calcium ionophore A23187 exhibited similar effects with triptolide which down-regulated AR expression in LNCaP cells (Figure 5). The results demonstrated that the down-regulation of AR protein by triptolide could depend on intracellular Ca^2+^ level.

## 4. Discussion

Although triptolide has been reported to be effective in PCa treatment, the mechanism of action is still unclear. Emerging studies have demonstrated that AR signaling plays a large role in the development and progression of PCa, even after castration [3,4]. Alteration of AR protein stability is one of the most important ways of regulating AR activity [13]. The present study showed that triptolide down-regulated AR expression in PCa LNCaP cells. In addition, when the synthesis of new AR was blocked by the protein synthesis inhibitor CHX, the treatment of triptolide also down-regulated AR protein level in LNCaP cell. The results demonstrated that AR protein stability was decreased by triptolide. Our previous investigation has showed that triptolide also block AR expression on transcriptional level through NFκB [11]. These results indicate that anti-PCa effects of triptolide may be reasonably attributed, at least partially, to its transcriptional and post-transcriptional regulation of AR.

In view of the important roles of protease in protein degradation, and calpains in AR protein hydrolyzation [14,15,16,22], we firstly focused on protease and calpains. Since PMSF, an inhibitor of serine and cysteine proteases which reacts with common proteases, could not inhibit the activity of calpain effectively [20], both PMSF and ALLM, a calpain inhibitor, were used in the current study. The observation that ALLM, but not PMSF, recovered the AR protein level decreased by triptolide, indicated that calpain activation could be involved in the down-regulation of AR protein by triptolide.

Several investigations suggested that calpains caused proteolytic elimination of AR and were beneficial for PCa treatment [14,15,16]. However, we found that the protein levels of two main calpains, including calpain-1 and calpain-2, as well as their endogenous inhibitory protein, calpastatin, were not noticeably altered by triptolide. This means that the calpain-dependent AR down-regulation by triptolide cannot result from the alteration of calpains or calpastatin protein levels. Then we further focused on calcium-triggered events, due to this negative data and the fact that calcium influx is known to be central in the regulation of calpain activities [21], as well as previous reports that the triptolide induces accumulation of Ca^2+^ in some cells [18,19]. These results indicate that, by increasing intracellular Ca^2+^ concentration, triptolide can activate calpain and thus break down the AR protein. In the presence of triptolide, the elevation of Ca^2+^ level was observed, and chelating Ca^2+^ with BAPTA-AM restored AR expression. The result further supported our assumption. Additional investigation is still needed to discover whether the activity of calpains were changed and which isoform of calpains can be involved in AR degradation caused by triptolide.

Apart from calpains, there are other pathways that can modulate AR protein stability. The most common form of AR degradation is carried out by the ubiquitin proteasome system [23]. However, triptolide inhibits proteasome activity in prostate and breast cancer [24], indicating that the ubiquitin proteasome pathway may be not responsible for AR protein degradation caused by triptolide [25]. In addition, although triptolide increases caspase-3 activity in PCa cells, only the polyglutamine-expanded form of AR is hydrolyzed by caspase-3 [25]. Cathepsin D also catalyzed AR degradation [26]. Cathepsins, which are non-caspase proteases, were also inhibited by ALLM [27]. Cathepsins may be activated by calpain, which indicates cathepsins were Ca^2+^ indirectly independent. Though the current investigation did not rule out the role of cathepsins in triptolide induced AR degradation, taking together the information, we suggest that calpain activation plays an essential role in the down-regulation of AR protein by triptolide.

In addition, it is worth noting that the Ca^2+^ concentration in the triptolide-treated group is lower compared with that of A23187 group, though the down-regulated AR level is similar in these two groups. AR mRNA is a significant contributor to AR protein level. Huang et al. [10] and our previous investigation [11] have shown that triptolide can regulate AR mRNA level. We have found that triptolide can down-regulate AR mRNA through the NFκB pathway [11]. Thus, it is reasonable to conclude that the down-regulation of AR protein level by triptolide may be through both Ca^2+^-dependent and Ca^2+^-independent pathways. However, quantitative investigation of the relative contribution of both pathways still needs further investigation. Moreover, though addition of A23187 following triptolide further down-regulated the level of AR protein, the concentration of A23187 employed in the current investigation also altered AR mRNA level [28]. To further illustrate the combined effect of A23187 and triptolide on AR protein, different compound concentrations or other investigation systems should be employed.

Androgen deprivation therapy is first-line treatment for PCa patients to impede the AR activity in metastasis PCa. However, gain-of-function mutations, in situ androgen production, androgen receptor gene amplification and AR splice variant have been reported to be responsible for the failure of androgen deprivation therapy [29]. It would be of greater importance to investigate whether triptolide regulated AR in androgen deprivation-resistant models. In addition, it has been reported that androgen level regulated AR mRNA level transcriptionally and post-transcriptionally [30]. Additional investigations using androgen-independent cell lines (such as 22RV1 and androgen-independent LNCaP subline cells) in androgen deprived medium are needed to further illustrate the clinical potential of triptolide in PCa treatment.

It is worth noting that the physiological functions of calpains are extensive, including regulation of apoptosis [31], cell migration [32], AR co-factor activation [33], and cell-to-cell communication via microparticle biogenesis [34,35] so on. Further exploration of the profound influence of triptolide on systematic function of cells or humans is still needed. In addition, although LNCaP cells are typical of conventional adenocarcinoma of the prostate [36], the explanation for the role of calpain activation and AR proteolysis caused by triptolide should be taken with caution, due to the limitation of data from only one cell line.

## 5. Conclusions

Together, these observations demonstrate that triptolide can activate calpain via promoting intracellular Ca^2+^ accumulation, and thus decrease the stability of AR protein, subsequently resulting in the breakdown of the AR protein in LNCaP cells. This work provides an experimental basis and evidence to elucidate the anti-PCa mechanisms of triptolide, a small-molecule natural product with great potential. However, further systematic studies are needed to acquire compelling support for triptolide as a potential drug for PCa.
